# Supplementary material for: Single neurons may encode simultaneous stimuli by switching between activity patterns
Source: Nat Commun. 2018 Jul 13;9:2715. doi: 10.1038/s41467-018-05121-8 (PMC6045601; doi:10.1038/s41467-018-05121-8)
Supplement: Supplementary file 1 — Supplementary Information [file 41467_2018_5121_MOESM1_ESM.pdf]

Single neurons may encode simultaneous stimuli by switching between activity patterns

Caruso V. C. et al.

Supplementary Tables

	Intermediate	Mixture	Outside	Single	Total
Wavy-Central+Symmetric	13 (5)	2			15 (5)
Wavy-Central	1				1
Wavy+Symmetric	4	2			6
Flat-Central+Symmetric	7 (3)				7 (3)
Flat-Central	2 (1)	1			3 (1)
Flat-Extreme+Skewed	8 (1)	12 (3)	19 (4)	46	85 (8)
Flat-Extreme+Symmetric	2	22 (19)		4	28 (19)
Flat-Extreme	3	31 (13)	1	16	51 (13)
Flat+Skewed	4 (2)				4 (2)
Flat+Symmetric	6	11 (4)		4	21 (4)
Flat	13 (2)	1		2	16 (2)
Extreme+Symmetric	1	4 (4)		1	6 (4)
Extreme		3 (2)		1	4 (2)
Central+Symmetric	9 (2)	1		2	12 (2)
Symmetric	36 (1)	42 (5)		14	92 (6)
Skewed	1 (1)				1 (1)
Ambiguous	5	2		3	10
Total	115 (18)	134 (50)	20 (4)	93	362 (72)

Supplementary Table 1. Complete listing of tags applied in the DAPP analysis. *Categories not shown (e.g. Wavy-Central+Skewed) did not receive any members. The numbers in parentheses are for triplets that produced a winning model in the whole trial analysis with a probability > 95%. The total is 362, since for one Single triplet, the DAPP analysis failed to compute the necessary metrics.*

Supplementary Figures

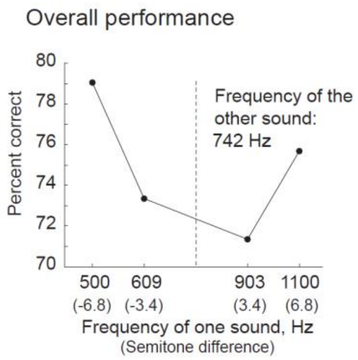

Supplementary Figure 1: Monkeys can localize two sounds.

Average accuracy for the dual-sound targets was overall over 70% correct. Performance was better for larger frequency separations. Data combined across all sessions in Data Set I.

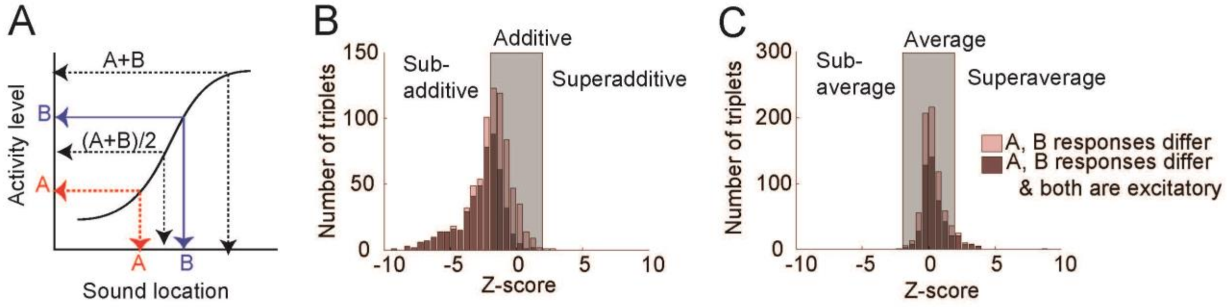

Supplementary Figure 2: Results of conventional analyses pooling across time and trials are consistent with averaging but not summation.

(A) *Schematic activity patterns of IC neurons in response to single-sounds A and B, and predicted response for dual sounds if the neuron sums inputs corresponding to individual sounds (A+B) or averages them ((A+B)/2).* ***(B)*** *Observed Z-scores of activity on dual-sound trials differ from the sum.* ***(C)*** *Observed Z-scores of activity on dual-sound trials correspond well to the average. The shaded areas indicate Z score values of +/- 1.96* standard deviations *(95% confidence intervals) of the sum (B) and of the average (C). This analysis was conducted on triplets of single- and dual-sound trials with a given set of locations and frequencies, pooling across intensities. Triplets were included if the single-sound responses differed (light bars, two-tailed t-test, $p<0.05$, $n=761$); results were similar when single-sound responses were different and both excitatory (dark bars, one-tailed t-test, $p<0.05$, $n=486$).*

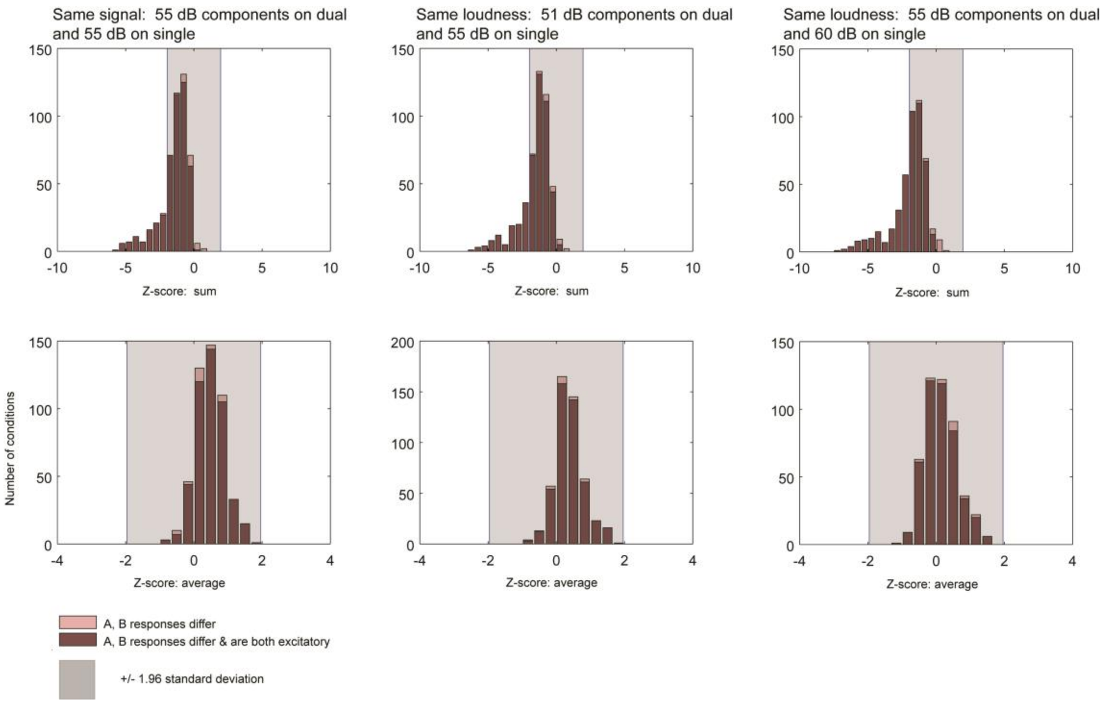

Supplementary Figure 3. Dual-sound responses do not depend on sound intensities

Same analysis as Supplementary Figure 2, but comparing dual-sound trials to single-sound trials that used the same signal sent to the speakers (left column), or for which the signal was amplified on single-sound trials to match the dual-sounds in loudness (middle and right columns). The results are essentially identical to each other.

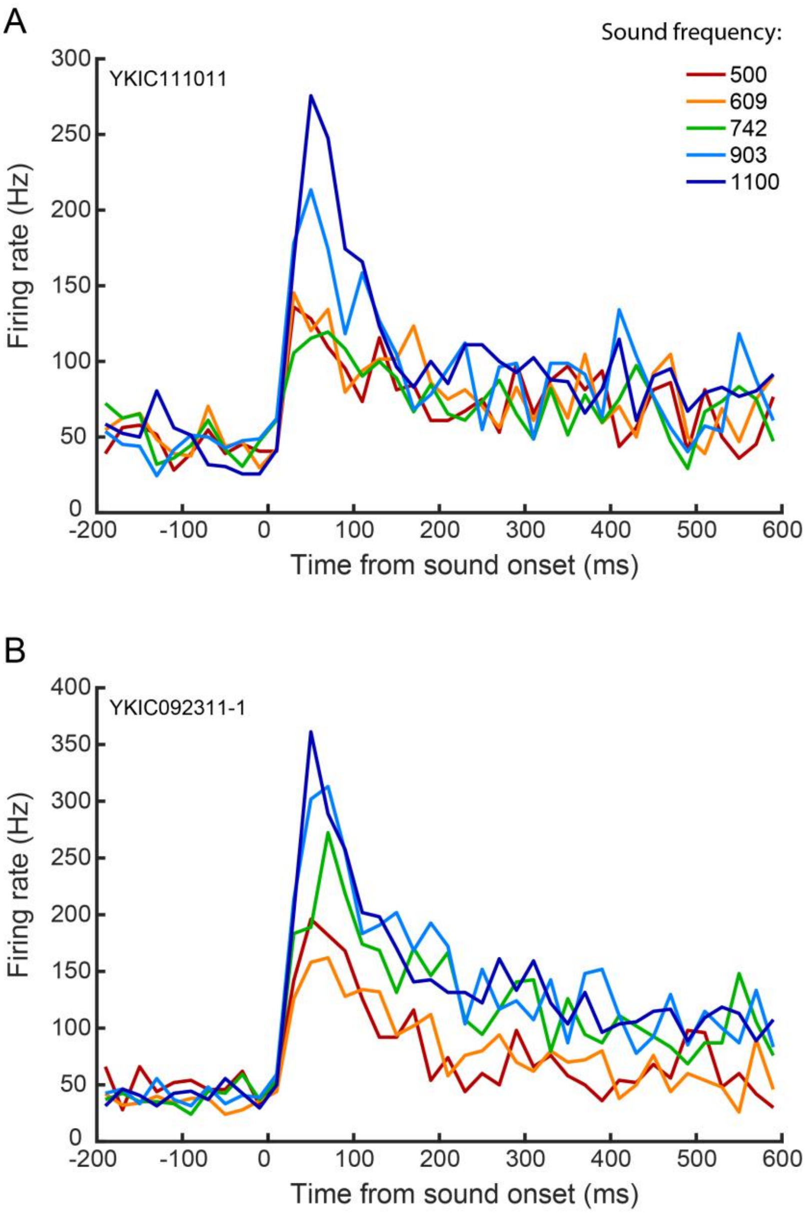

Supplementary Figure 4. *Frequency responses of the example cells in the main text. For each cell, the average firing rate is computed across single-sound trials with the same central frequency but pooling across speaker locations (20ms bins with no smoothing). (A) Same cell as Figure 1E, 2E (B) Same cell as Figure 1F, 2G, 3C.*

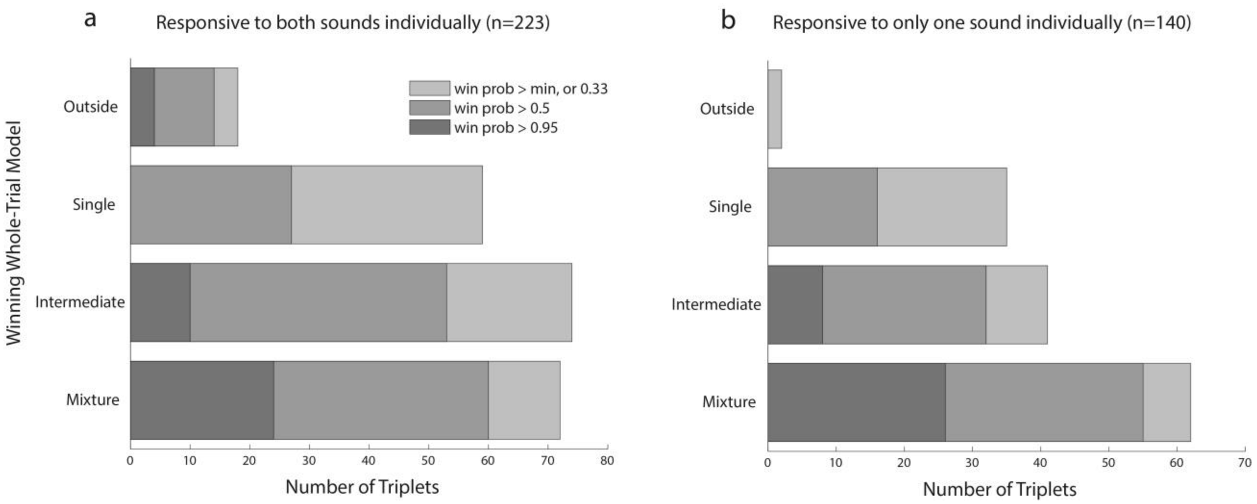

Supplementary Figure 5. Whole-trial analysis results broken down by whether neurons were responsive to both of the sounds in the triplet when presented individually (a) or only one of the two sounds (b). *Results were largely similar except that Outsides were more common when the neuron was responsive to both sounds. Responsiveness was determined by comparing the firing rate in the 600 ms period after target onset to the 500 ms period prior to sound onset (t-test, p<0.05).*

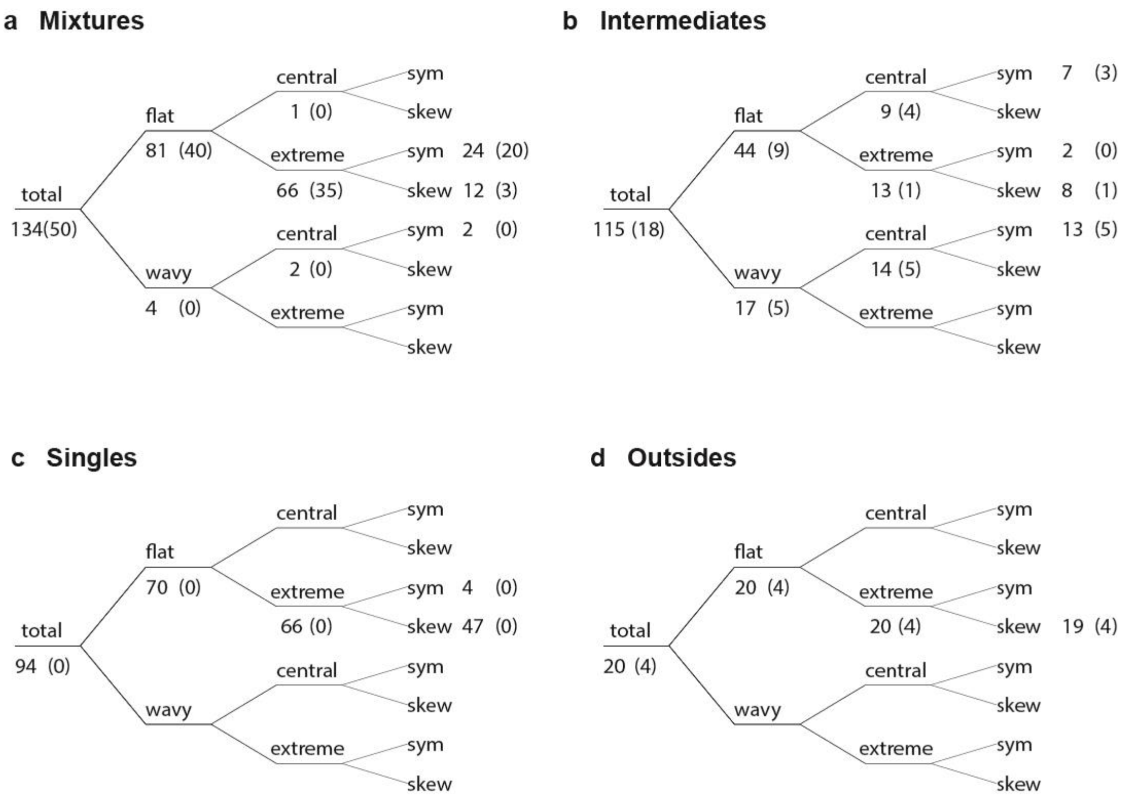

Supplementary Figure 6. Hierarchical depiction of the DAPP tags*, considering first Flat vs. Wavy, then Central vs. Extreme, then Symmetric vs. Skewed, as a function of whole trial model classification: (a) Mixture, (b) Intermediate, (c) Singles, (d) Outside. As in Supplementary Table 1, numbers in parentheses are for triplets that produced a winning model in the whole trial analysis with a probability > 0.95 and the total included here is 362. For clarity, labels "0(0)" are not shown.*

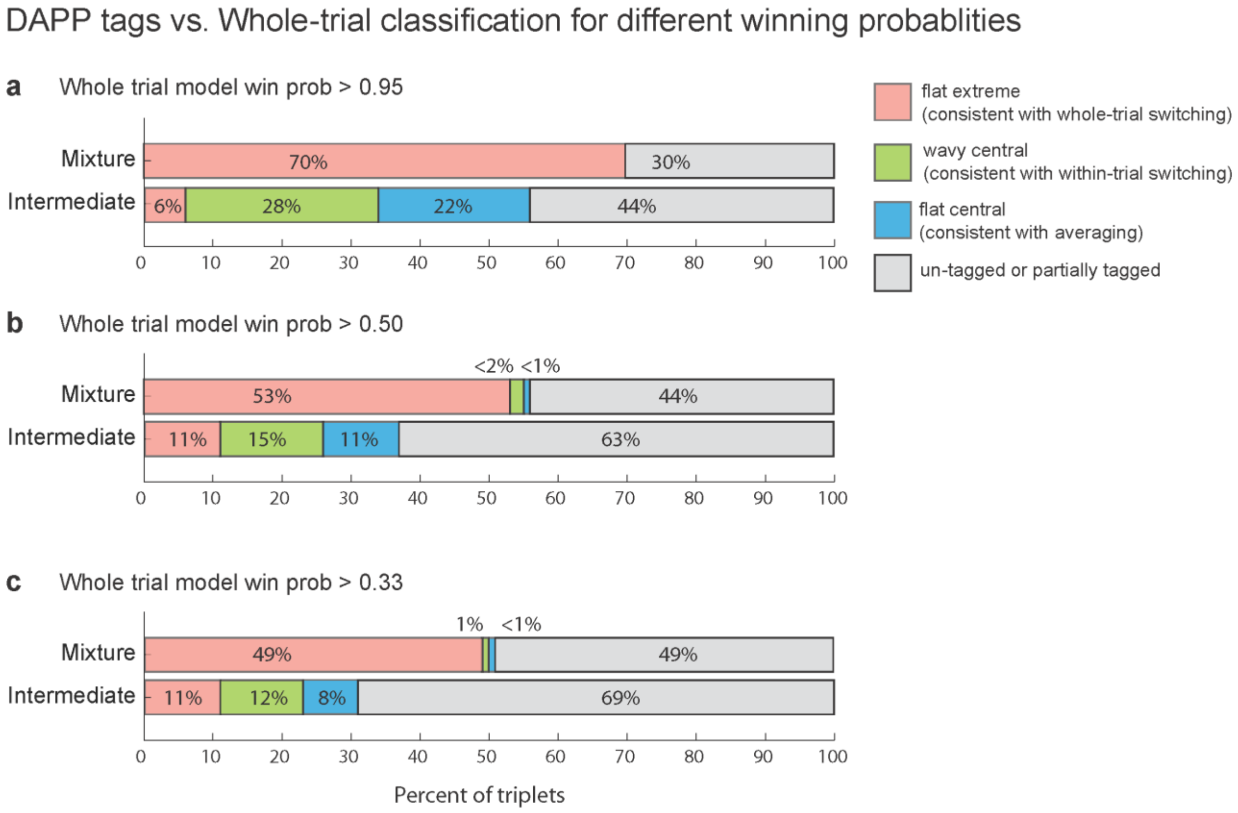

Supplementary Figure 7. *Relationship between the three relevant DAPP tag combinations (Flat-Extreme, Wavy-Central, and Flat-Central) and whole-trial Poisson classification, for different levels of winning probability for the whole-trial analysis. (a) Winning probability greater than 0.95 (this panel is identical to Figure 3E in the main text). (b) Winning probability greater than 0.50. (c) Winning probability larger than 0.33.*

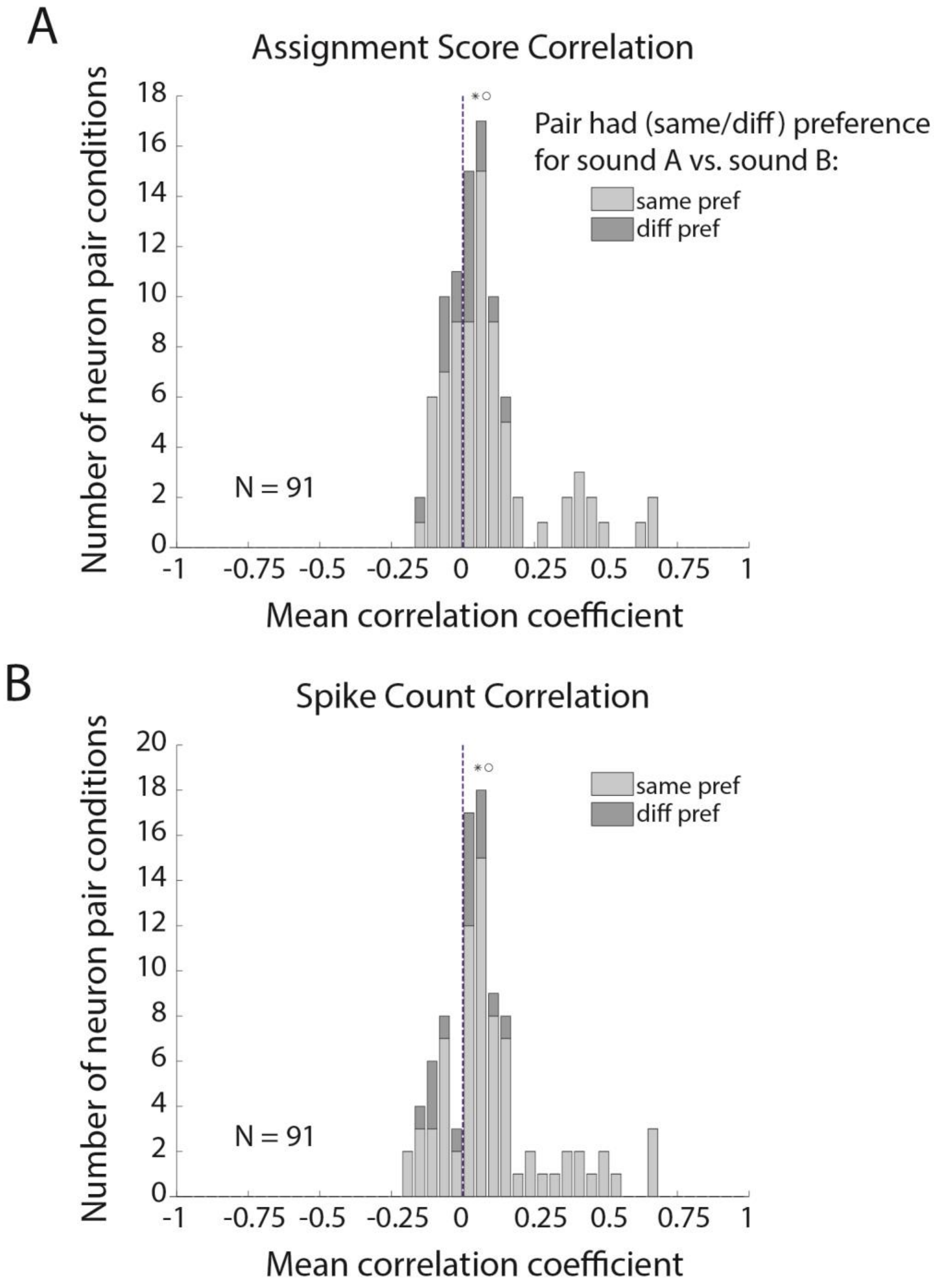

Supplementary Figure 8. *A. Same analysis as in Figure 4B, but shaded according to whether both neurons in the pair preferred the same sound (e.g.. A_{resp}> B_{resp} or vice versa for both neurons in the pair; light gray) vs. different sounds (e.g. A_{resp}> B_{resp} for one neuron and A_{resp}< B_{resp}. for the other; dark gray). B. The same analysis as panel A, but using spike counts in each bin instead of A vs. B assignment scores. Asterisks indicate the median; circles indicate the mean.*
